# Bisphenol A and Bisphenol S Oxidative Effects in Sheep Red Blood Cells: An *In Vitro* Study

**DOI:** 10.1155/2021/6621264

**Published:** 2021-03-24

**Authors:** E. Baralla, M. P. Demontis, M. V. Varoni, V. Pasciu

**Affiliations:** Department of Veterinary Medicine, University of Sassari, Sassari, Italy

## Abstract

Bisphenols (BPs) are plastic components widely used worldwide and occurring in the environment. Exposure to these compounds is known to be harmful for animals and humans at different levels. The aim of this study was to evaluate and compare the oxidative effects of bisphenol A (BPA) and bisphenol S (BPS) in sheep. Reactive oxygen species (ROS) production and correlated structural alterations in sheep erythrocytes were investigated *in vitro*. Blood samples from four ewes were collected at fasting from the jugular vein using vacuum collection tubes containing EDTA. For ROS assay in erythrocytes, blood was properly diluted and BPA or BPS was added to obtain final bisphenol concentrations in the range between 1 and 300 *μ*M. 2′,7′-Dichlorodihydrofluorescein diacetate (H2DCF-DA) 3 *μ*M was added to the samples, and fluorescence was read in four replicates using a microplate reader. To evaluate erythrocyte shape, blood smears of blood treated with the different concentrations of BPS and BPA were prepared. A significant increase in ROS production was observed when concentrations of BPS and BPA increased from 1 to 100 *μ*M (*p* < 0.05). At the higher concentrations of the two studied BPs (300 *μ*M of BPS and 200-300 *μ*M of BPA), a ROS decrease was observed when compared to the control group (*p* < 0.01). Erythrocytes' shape alterations were observed in cells treated with BPS and BPA 200-300 *μ*M 4 hours after the beginning of the treatment. This study confirms that BPA and BPS exhibit oxidative effects on sheep erythrocytes. At higher concentrations, BPA was able to modify erythrocytes' shape, while BPS altered their membrane as a sign of a protein clustering that could lead to eryptosis. These BPs' effects are consequent to intracellular ROS increase.

## 1. Introduction

Bisphenol A (BPA) is an endocrine disruptor that has been widely used in the world with several uses like in the manufacturing of plastics, epoxy resins, and a variety of plastic and paper consumer products and food [1–3]. There are many concerns about its well-known endocrine disrupting action as well as about its hepatotoxic, neurotoxic, and carcinogenic effects on the human body. Several studies report that these BPA toxic effects are also associated with the production of ROS [4, 5], and sometimes, antioxidants' use can prevent these effects [6]. A significant increase in the levels of intracellular ROS production and a decrease of antioxidant capacity was found in KGN cells (a granulosa-like tumor cell line) after treatment with high concentrations of BPA and its analogues [7]. BPA and the products of its metabolism can reach the body and, accordingly, the blood, through the assumption of drinking water and food and through dental material [8–12]. Due to the toxicity of this compound, and to comply with restrictions and regulations, manufacturers have progressively replaced it with substitutes. One of the main substitutes used is bisphenol S (BPS). However, BPS has become so common in society that it was determined in urine samples of 81% of people in the United States and Asia [13]. Therefore, over the last few years, there has been a considerable scientific effort to evaluate the safety of BPS, since most people and animals are exposed to it through different routes. Unfortunately, several studies report that BPS has similar androgenic/estrogenic effects in different experimental models [14, 15].

The extended use of BPs has led to high environmental exposure of these compounds [16]. Moreover, given the widespread use of BPs, they are ubiquitous in the environment, and their accumulation can be dangerous not only for humans but also for animals that, through different routes, can come in contact with them. Plastics are in fact widely used in agriculture and animal breeding for a variety of purposes like covering for crops and wrappings for hay bales.

BPA and its analogues can come into interaction with plasma proteins, hemocytes, and, first of all, with erythrocytes as carriers for xenobiotics. Being the most plentiful cells in the circulatory system, erythrocytes are responsible for transporting oxygen and other chemicals; thus, they are strongly exposed to xenobiotics like bisphenols (BPs) [12]. Maćczak et al. (2015) reported that BPA, bisphenol F (BPF), BPS, and bisphenol AF (BPAF) exhibited different hemolytic and oxidative potentials and caused morphological changes in red blood human cells.

The aim of our study was to investigate the effects of BPs in sheep through an *in vitro* study in ewes' erythrocytes. To avoid pain and permanent danger to animals, the *in vitro* approach permits to predict risks related to the environment and animals together with the application of the 3Rs principle, Reduce, Refine, Replace animal use, in accordance with the Directive 2010/63 EU. The sheep model represents a valuable tool in studying the effects of xenobiotics on animals and humans also at different development stages [17]. As an example, sheep was used to help understand BPs' effects and mechanism of action in a pregnancy model [18–20]. Another author used sheep blood for his work, and in particular, he studied sheep blood contaminated with BPA that was detected in both plasma and red blood cells (RBC) (10 times lower than in plasma), indicating that BPA could have migrated from plasma into RBC [21]. Guignard et al. (2016) reported that part of the BPA ingested by sheep could be directly absorbed by passive diffusion through the buccal mucosa due to the molecule's physicochemical properties [22]. In this way, BPA directly attained the systemic circulation avoiding the hepatic first-pass effect. Furthermore, sheep thoroughly masticates its food, thus ensuring a long enough contact of the contaminated food with the buccal mucosa. In this animal model, a dual mechanism of absorption was observed. In fact, the mucosal absorption during food chewing was followed by the one from the gastrointestinal tract. This dual mechanism might therefore be associated with higher bioavailability and higher blood concentrations than routes of administration limited to intestinal absorption as reported by pharmacokinetic studies [23, 24].

Environmental contaminants can interfere with physiological systems and ruminants' capacity to reproduce and fight diseases, especially when animals are grazed on contaminated pastures or drinking water. In light of this, understanding the effects of pollutants requires knowledge at the cellular and molecular levels.

Given these premises, in this work, we report a preliminary study to evaluate the oxidative effect produced by BPs on sheep erythrocytes. In particular, we compared the effect of BPA and BPS on ROS production and related structural alterations *in vitro* in the cellular model described.

## 2. Materials and Methods

All procedures involving animals in this study were approved by the Local Animal Care and Use Committee (authorization code: 2899 of 17/01/2018). Ewes were confined outdoors with access to a sheltered area, at the experimental facilities of the Department of Veterinary Medicine at the University of Sassari, Italy (40°43′40.33^″^N, 8°33′1.33^″^E). Blood samples from four healthy ewes were collected at fasting (07:00 a.m.) from the jugular vein using 10 mL vacuum collection tubes containing EDTA K2 (Vacutainer Systems Europe; Becton Dickinson, Meylan Cedex, France). A pool of blood was used for the analysis. The mean concentration of sheep erythrocytes was 12∗10^6^ cells/*μ*L.

Blood was diluted 1 : 50 with Hanks' Balanced Salt Solution (HBSS) and used for the treatment with BPA and BPS for hemolysis test and for blood smear preparation. For ROS assay, a further dilution was made as described below. BPA and BPS stock solutions were prepared in methanol.

### 2.1. ROS Assay

For the measurement of ROS production in erythrocytes, blood was further diluted 1 : 4 using HBSS (total dilution 1 : 400), adding BPA or BPS at different concentrations (1, 10, 100, 200, and 300 *μ*M) and 2′,7′-dichlorodihydrofluorescein diacetate (H_2_DCF-DA) at the final concentration of 3 *μ*M. Within the cell, the esterases cleave the acetate groups on H_2_DCF-DA, thus trapping the reduced form of the probe 2′,7′-dichlorodihydrofluorescein (H_2_DCF). Intracellular ROS oxidize H2DCF, yielding the fluorescent product, DCF. Fluorescence was measured using a FLUOstar Omega microplate reader (BMG LABTECH). Excitation and emission wavelengths used for fluorescence quantification were 485 and 535 nm, respectively. All fluorescence measurements (RFU = Relative Fluorescence Unit) were corrected for background fluorescence. Treatments for 4 hours with BPs induced variation of fluorescence versus control (CTRL) (cells without BPs treated with methanol) and were measured in four replicates. Data were expressed as means ± SD [25, 26].

### 2.2. Preparation of Blood Smears

Diluted blood was incubated at 37°C for 4 hours with different concentrations of BPS and BPA (1, 10, 100, 200, and 300 *μ*M). After incubation, blood samples were used to prepare blood smears. A slide for each concentration of BP was viewed at high magnification using an inverted system microscope (Olympus X71 model TH4200). An untreated blood smear was used as the control.

### 2.3. Hemolysis

After incubation at 37°C for 4 hours with BPA and BPS concentrations ranging from 1 to 300 *μ*M, blood samples were gently centrifuged (100 g for 3 min), and the supernatant was used for hemolysis test. The absorbance of the hemoglobin in the supernatant was read at 405 nm. Data were expressed as % means ± DS. The absorbance value in the supernatant of sheep RBC lysed in H_2_O was considered as 100% hemolysis [27].

### 2.4. Statistical Analysis

Statistical Analysis Data are expressed as mean ± SEM of at least four replicates. Results were analyzed by a monofactorial ANOVA (Minitab® 18.1). Statistical significance was accepted at *p* < 0.05.

## 3. Results

### 3.1. ROS Assay

**Figure 1:**
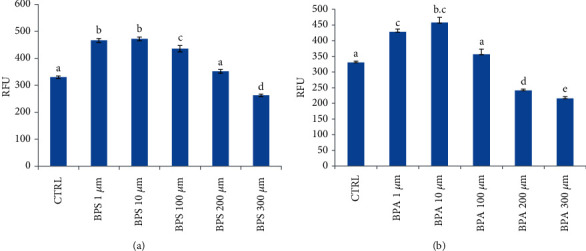
ROS production in sheep erythrocytes treated with BPS (a) and BPA (b) (1-300 *μ*M). All fluorescence measurements (RFU) after 4 hours of treatment were corrected for background fluorescence, and excitation and emission wavelengths were 485 and 535 nm, respectively. CTRL were cells treated with methanol. Statistical Analysis Data are expressed as mean ± SEM of at least 4 replicates. Results were analyzed by a monofactorial ANOVA (Minitab® 18.1). Statistical significance was accepted at *p* < 0.05.

ROS production in sheep erythrocytes treated for 4 hours with BPA and BPS at concentrations ranging from 1 *μ*M to 300 *μ*M was determined. As shown in Figure 1, a significant increase in ROS production was observed when concentrations of BPS and BPA increased from 1 to 100 *μ*M (*p* < 0.01 for 1 and 10 *μ*M and *p* < 0.05 for 100 *μ*M) (Figure 1(a)) and from 1 to 10 *μ*M (*p* < 0.01 for 1 *μ*M and *p* < 0.05 for 10 *μ*M) (Figure 1(b)), respectively, when compared to the control group. Furthermore, at the higher concentrations of the two studied BPs (300 *μ*M of BPS and 200-300 *μ*M of BPA), a ROS decrease was observed when compared to the control group (Table 1). In RBC treated with BPS at the concentration of 200 *μ*M (Figure 1(a)) and BPA at the concentration of 100 *μ*M (Figure 1(b)), ROS production was reported to values similar to that of the control group.

### 3.2. Blood Smears

Blood smears of erythrocytes treated for 4 hours with the tested BP concentrations were performed. They were read in order to evaluate possible structural changes in the shape of RBC when compared to blood smears of erythrocytes of the control group.

**Figure 2:**
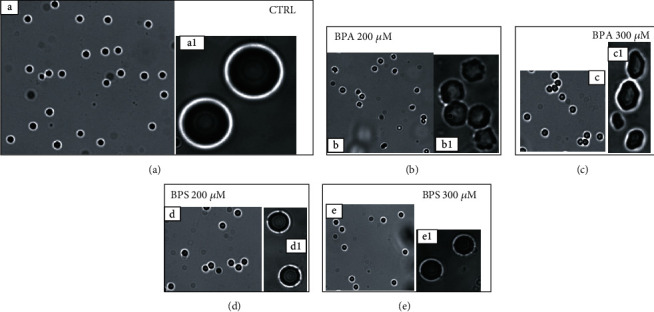
Smears of sheep's blood: shape of erythrocytes treated with methanol (CTRL) ((a) 10x and (a1) 100x) with BPA 200 *μ*M ((b) 10x and (b1) 100x), BPA 300 *μ*M ((c) 10x and (c1) 100x), BPS 200 *μ*M ((d) 10x and (d1) 100x), and BPS 300 *μ*M ((e) 10x and (e1) 100x), 4 hours after the beginning of the treatment.

Erythrocytes' shape alterations were observed in cells treated with BPS and BPA 200-300 *μ*M 4 hours after the beginning of the treatment (Figure 2). RBC treated for 4 hours with other concentrations of BPs investigated in this work did not show any shape alteration.

### 3.3. Hemolysis

**Figure 3:**
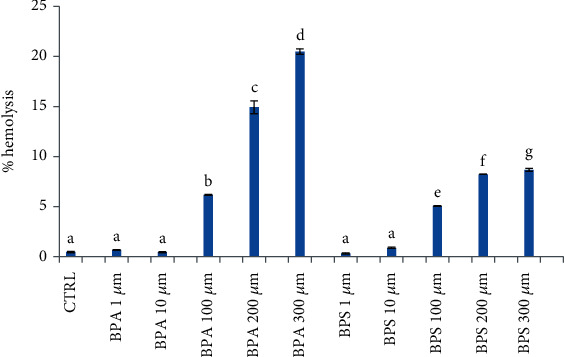
RBC hemolysis percentage after incubation with BPA and BPS at concentrations of 1, 10, 100, 200, and 300 *μ*M; CTRL were cells treated with methanol. Statistical Analysis Data are expressed as mean ± SEM of at least 4 replicates. Results were analyzed by a monofactorial ANOVA (Minitab® 18.1). Lowercase letters indicate significant differences between groups (*p* < 0.01).

After incubation or sheep RBC with BPs, a significant hemolysis increase was observed with increasing BPA and BPS concentrations (from 100 to 300 *μ*M) (*p* < 0.01; Figure 3), with a major hemolysis % observed in RBC treated with BPA.

## 4. Discussion

BPs' wide use has led them to become ubiquitous in the environment, causing several harmful effects for human and animal health. Furthermore, the exposure of humans and animals to these chemicals can be chronic, from the prenatal stage to adulthood. BPs are indeed constituents of daily used products, and the contact with them can be either indirect or direct [28]. The study of the effects of chemicals such as BPs can be favorably developed on erythrocytes, given that RBC are relatively easy to obtain, can be used for *in vitro* or *in vivo* experiments, and are sensitive biomarkers of chemical-induced damage. Maćczak et al. (2015, 2017) investigated for the first time the effects of BPA and some of its analogues on the oxidative stress system in human erythrocytes, confirming their ability to induce oxidative changes in this nonnucleated cells, also if they are considered to be more resistant to oxidative stress than nucleated ones. In another study, the same authors reported that BPA and its analogues caused changes in cytosolic calcium ion levels in human erythrocytes after 4 hours of treatment [12]. Other literature data revealed that eryptotic changes are associated with the increase of intracellular calcium ions in RBC. Moreover, it was found that eryptosis was triggered by H_2_O_2_ and paralleled by increased ROS and [Ca^2+^] and that erythropoietin, which possesses antioxidant property, could protect erythrocytes against oxidative stress-induced eryptosis [29]. It was also reported that alterations in intracellular ROS caused eryptosis, along with other modifications like alterations of calcium ion levels, caspase-3, calpain activation, and phosphatidylserine translocation in this cell type [30]. At the moment, no study is reported in the literature investigating the oxidative effects of BPs in sheep erythrocytes. Nevertheless, sheep is an excellent animal model to study several physiological or pathological conditions [18]. Moreover, given the high accumulation in the environment of plastic products [31], livestock can be directly exposed to many emerging contaminants like BPs. Furthermore, direct oral absorption of BPs during food chewing in the buccal mucosa, might be associated with higher bioavailability and higher blood concentrations of this chemical in sheep [22].

In this ruminant, exposure to BPs can also occur though drinking water, contaminated food, and ingestion of soil. Moreover, animal feed and water can be stored in plastic containers that, with the help of the high temperatures reached in certain seasons, can contribute to the release of BPs, representing a threat for these domestic ruminants.

In this work, we studied ROS production *in vitro* in sheep erythrocytes, treated with different BPA and BPS concentrations (1-300 *μ*M) for 4 hours. We observed an increase in ROS production at increasing concentrations of BPA (1-10 *μ*M) and BPS (1,10,100 *μ*M). At the higher concentrations of the two studied BPs (200 and 300 *μ*M of BPA and 300 *μ*M of BPS), ROS production decreased when compared to control (Figure 1). This decreasing condition could be explained probably because the oxidative damage was so great as to trigger hemolysis, which in vivo can result in eryptosis, as reported in the literature [12, 29, 30]. This aspect was confirmed by the observed alteration of erythrocytes treated with BPS and BPA 200-300 *μ*M 4 hours after the beginning of the treatment (Figure 2). Membrane erythrocytes' alteration observed in this study after treatment with BPA at the highest concentration used confirms results obtained by Maćczak et al. (2015). The same authors did not observe any RBC shape's modification when cells were treated with BPS at concentrations also higher than those used in our study. Nevertheless, in this work, erythrocytes treated with BPS 300 *μ*M did not show any shape modification in line with Maćczak et al. (2015), but as shown in Figure 2, alterations in membrane integrity were observed.

Several studies report that the accumulation of oxidative damage in erythrocytes leads to irreversible clustering of integral membrane protein band 3. This clustering is an essential process of RBC senescence and eryptosis that can result in cellular removal [32–35].

Under in vitro conditions, the damaged erythrocytes cannot be eliminated and they undergo hemolysis [27] as observed in our study (Figure 3). In particular, high BPA concentrations caused a higher % of hemolysis when compared to its analogue BPS. This trend is in accordance with what the above described for shape and membrane integrity RBC alterations.

Many studies evaluate several harmful effects of BPS [14, 36–38] like oxidative stress, and results found in this study are in line with them. Indeed, our results confirm that, also if BPS is considered as a safer alternative to BPA, it is able to exhibit oxidative effects on sheep erythrocytes also if to a lesser extent than its regulated analogue as reported also by Maćczak et al. (2017) for human erythrocytes.

## 5. Conclusions

At the moment, BPs' impact studies are relatively few in sheep, and results obtained in this work show that BPs are able to exert oxidative effects in erythrocytes of this livestock animal. Given that BPs are ubiquitous in the environment and have a wide range of toxicity profiles, these findings highlight the potential adverse effects of BP exposure which should require further interest from competent authorities.

## Figures and Tables

**Table 1:** % in ROS production in erythrocytes increases significantly when compared to control. Statistical Analysis Data are expressed as mean ± SEM of at least 4 replicates. Results were analyzed by a monofactorial ANOVA (Minitab® 18.1).

Bisphenols	Bisphenols concentrations	% ROS production vs. CTRL
BPS	1 *μ*M	+29.2 ± 7.1 ↑^∗∗^
	10 *μ*M	+30 ± 6.9 ↑^∗∗^
	100 *μ*M	+24.2 ± 11.9 ↑^∗^
	200 *μ*M	+6 ± 6.5
	300 *μ*M	↓^∗∗^−20 ± 4.49

BPA	1 *μ*M	+22.8 ± 8.8 ↑^∗∗^
	10 *μ*M	+28^∗^ ± 16.8 ↑^∗^
	100 *μ*M	+7 ± 5
	200 *μ*M	↓^∗∗^−26.9 ± 4.22
	300 *μ*M	↓^∗∗^−34.65 ± 5.82

↑^∗^ statistically significant increment (*p* < 0.05); ↑^∗∗^ statistically significant increment (*p* < 0.01); ↓^∗∗^ statistically significant decrement (*p* < 0.01).

## Data Availability

Data used to support the findings of this study are included within the article.
